# Cause of Consumption

**Published:** 1876-04

**Authors:** 


### CAUSE OF CONSUMPTION.

Not by bad colds, nor hereditary predisposition, nor drinking liquor, nor tight lacing—for men do not lace, and yet as many of them die of consumption as women; few habitual drinkers die of that disease; and as for hereditary taint and bad colds, millions of the latter have gotten well of themselves, while the naturally feeble are compelled to an habitual carefulness of themselves, which gives them, in multitudes of cases, an immunity against all disease, except that of old age.

The very essence of consumption is a decline in flesh. Flesh is made of the food we eat; if that food does not give flesh, does not sustain the proper proportion of it, we begin to fade, and fail, and consume away.

But as there is not one in a hundred thousand who has not a plenty of food, and yet one out of every nine in the Union dies of consumption every year, the cause of that malady is not a want of food, although it is a want of flesh; and yet only food can give flesh. It must then be from the fact, that although we have a plenty of food, that food does not give the amount of flesh and strength which it ought to. The process by which food gives flesh is a double one—digestion and assimilation; in other words, it is the taking of the nourishment from the food, and distributing it to the body at various points.

The human body is much like a clock with its many wheels; if one goes slow the others go slow, and bad time is the result; if one little wheel of the body (one organ or one gland) works imperfectly or slowly, all the others are influenced thereby, and lag also. But what is the wheel which oftenest gets out of gear? It is the liver. What infallible telegraphic signal is always made when the liver is out of order? It is constipation of the bowels. In a natural healthful state of the human body, the bowels act at least once a day; less than that is a certain indication that the liver is halting in its pace, and if the admonition is allowed to remain long unheeded, disease is as inevitable as the falling of a stone when cast from the hand. The moment constipation commences, that moment the blood begins to become impure and poor; loses its life and heat, and the body chills; "the least thing in the world" causes a chill to run along the back, or gives a cold outright; and a cold being so easily contracted, before one is cured another comes on, and that cold is continued, and this is the synonym of consumption! This article might therefore be closed with the important practical inference, that by avoiding or correcting constipation, very many of those diseases might be avoided or cured, which arise from impure blood. But another step may be taken with great advantage. What makes the liver grow slow in its action? what makes it torpid or work weakly?

For the same reason that an over-worked horse, or servant, or man, becomes slower and slower in every motion.

The liver has a certain amount of bile to manufacture every day; this bile is made out of the blood; if that blood be of a good quality every day, the work is regularly performed and well done, for the space of an hundred years! Any mechanic knows that it is a comparatively easy matter to make a good job out of good materials; but to turn out a good day's work from bad materials is a most tiresome, wearing, wasting thing. The blood becomes of a bad material within six hours after a man eats too much; if that excess is committed three times a day, this bad blood becomes a permanent supply in time; the liver for a while does its duty; longer, according to the greater vigor of the constitution; but sooner or later it lags; it is worked to death. In the mean while, constipation becomes a habit, and the work of death is done. But this curious fact is not unfrequent: when consumption is fastened on the lungs by continued colds, all the disease of the body is in a measure attracted there, the liver resumes its apparent healthy function, and the bowels remain daily acting, until death.

Over-eating, then, three times a day, may be considered as a primary, a radical cause of the great majority of consumptive diseases, and each reader is advised to take the matter in hand as to himself, by

1. Eating moderately every day.
2. By securing a daily action of the bowels.

But if he is so much of a baby—has so little self-denial and manly moral courage, that he "*can't help eating too much!*" then an antagonizer of hearty eating is presented. Work steadily in the open air every day, from sunrise until sunset, with dry feet and dry clothing, singing or whistling all the time.

As proof that a free open air exposure is a preventive of consumption, it seems to be conceded that fewer persons die in the South of that disease than in the North, among an equal population; the mildness of the weather, and the leisure habits of the people, invite and allow an out-door life during all the hours of daylight. One person out of every five dies of consumption in New-England; one in thirty-six in Georgia. We

